# Supplementary material for: Individual variability in behavior and functional networks predicts vulnerability using an animal model of PTSD
Source: Nat Commun. 2019 May 30;10:2372. doi: 10.1038/s41467-019-09926-z (PMC6543038; doi:10.1038/s41467-019-09926-z)
Supplement: Supplementary file 2 — Reporting Summary [file 41467_2019_9926_MOESM2_ESM.pdf]

nature research

Corresponding author(s): Nanyin Zhang

Reporting Summary

Nature Research wishes to improve the reproducibility of the work that we publish. This form provides structure for consistency and transparency in reporting. For further information on Nature Research policies, see Authors & Referees and the Editorial Policy Checklist.

Statistical parameters

When statistical analyses are reported, confirm that the following items are present in the relevant location (e.g. figure legend, table legend, main text, or Methods section).

n/a	Confirmed	
☐	☒	The exact sample size (n) for each experimental group/condition, given as a discrete number and unit of measurement
☐	☒	An indication of whether measurements were taken from distinct samples or whether the same sample was measured repeatedly
☐	☒	The statistical test(s) used AND whether they are one- or two-sided<br>*Only common tests should be described solely by name; describe more complex techniques in the Methods section.*
☐	☒	A description of all covariates tested
☐	☒	A description of any assumptions or corrections, such as tests of normality and adjustment for multiple comparisons
☐	☒	A full description of the statistics including central tendency (e.g. means) or other basic estimates (e.g. regression coefficient) AND variation (e.g. standard deviation) or associated estimates of uncertainty (e.g. confidence intervals)
☐	☒	For null hypothesis testing, the test statistic (e.g. F, t, r) with confidence intervals, effect sizes, degrees of freedom and P value noted<br>*Give P values as exact values whenever suitable.*
☒	☐	For Bayesian analysis, information on the choice of priors and Markov chain Monte Carlo settings
☒	☐	For hierarchical and complex designs, identification of the appropriate level for tests and full reporting of outcomes
☒	☐	Estimates of effect sizes (e.g. Cohen's d, Pearson's r), indicating how they were calculated
☐	☒	Clearly defined error bars<br>*State explicitly what error bars represent (e.g. SD, SE, CI)*

Our web collection on statistics for biologists may be useful.

Software and code

Policy information about availability of computer code

Data collection	All imaging data were collected using paravision 6.0
Data analysis	Imaging data were processed using custom Matlab (2018) code. Behavioral data were analyzed using ANY-maze (Stoelting Co., Reston, VA)

For manuscripts utilizing custom algorithms or software that are central to the research but not yet described in published literature, software must be made available to editors/reviewers upon request. We strongly encourage code deposition in a community repository (e.g. GitHub). See the Nature Research guidelines for submitting code & software for further information.

Data

Policy information about availability of data

All manuscripts must include a data availability statement. This statement should provide the following information, where applicable:

- Accession codes, unique identifiers, or web links for publicly available datasets
- A list of figures that have associated raw data
- A description of any restrictions on data availability

Data supporting the findings of this manuscript are available from the corresponding author upon reasonable request. Source data underlying Figs. 1b–d, 2b–d, 3 and 5, as well as Supplementary Figs. 1, 2, 3, 4c, 5a, 5c–e, and 6b are provided as a Source Data file.

Field-specific reporting

Please select the best fit for your research. If you are not sure, read the appropriate sections before making your selection.

☒ Life sciences ☐ Behavioural & social sciences ☐ Ecological, evolutionary & environmental sciences

For a reference copy of the document with all sections, see nature.com/authors/policies/ReportingSummary-flat.pdf

Life sciences study design

All studies must disclose on these points even when the disclosure is negative.

Sample size	The sample size for each experiment was reported. The main cohort of animals included 87 rats, in which 23 rats were controls and 64 rats were exposed to predator scent using a single-episode predator scent exposure in an inescapable environment paradigm.
Data exclusions	Two rats were removed from the exposed group before analysis due to health concerns.
Replication	We used different behavioral tests and neuroendocrine measure to confirm the validity of our findings.
Randomization	The initial group allocation was completely random.
Blinding	Grouping was based on behavioral test results, which were obtained by the computer program (AnyMAZE). Experimenters did not have the information of the group of each animal before that.

Reporting for specific materials, systems and methods

Materials & experimental systems

n/a	Involved in the study
☒	☐ Unique biological materials
☒	☐ Antibodies
☒	☐ Eukaryotic cell lines
☒	☐ Palaeontology
☐	☒ Animals and other organisms
☒	☐ Human research participants

Methods

n/a	Involved in the study
☒	☐ ChIP-seq
☒	☐ Flow cytometry
☐	☒ MRI-based neuroimaging

Animals and other organisms

Policy information about studies involving animals; ARRIVE guidelines recommended for reporting animal research

Laboratory animals	Adult Rats, Long Evans
Wild animals	n/a
Field-collected samples	n/a

Magnetic resonance imaging

Experimental design

Design type	resting-state fMRI
Design specifications	n/a
Behavioral performance measures	n/a

Acquisition

Imaging type(s)	functional
Field strength	7T
Sequence & imaging parameters	Single-shot echo planar imaging
Area of acquisition	whole brain

Diffusion MRI ☐ Used ☒ Not used

Preprocessing

Preprocessing software	rsfMRI images were preprocessed by performing alignment using Medical Image Visualization and Analysis (MIVA, http://ccni.wpi.edu/), all subsequent processing was performed using matlab.
Normalization	Data from all rats were normalized to a standard atlas using linear transformation.
Normalization template	The template was based on Swanson Atlas
Noise and artifact removal	Noise and artifact removal include motion correction using SPM 12 (http://www.fil.ion.ucl.ac.uk/spm/), spatial smoothing (Gaussian kernel: FWHM = 0.75 mm) and temporal smoothing (band-pass filter, cutoff frequencies: 0.01-0.1Hz), and regression of motion parameters and signals from the white matter and ventricles.
Volume censoring	EPI volumes with relative framewise displacement (FD) > 0.2 mm and their immediate temporal neighbors were first removed. In addition, the first 10 volumes of each rsfMRI run were also removed to ensure the magnetization was at steady state. rsfMRI runs with > 20% volumes removed were excluded from further analysis.

Statistical modeling & inference

Model type and settings	Linear correlation.
Effect(s) tested	We tested the correlation between resting-state functional connectivity in each connection and freezing time across all exposed animals.

Specify type of analysis: ☒ Whole brain ☐ ROI-based ☐ Both

Statistic type for inference (See Eklund et al. 2016)	The statistical test was conducted on each connection.
Correction	Multiple comparisons were corrected using false-discovery rate (FDR) correction.

Models & analysis

n/a	Involved in the study
☐	☒ Functional and/or effective connectivity
☒	☐ Graph analysis
☒	☐ Multivariate modeling or predictive analysis

Functional and/or effective connectivity	Functional connectivity analysis was conducted using region-of-interest (ROI) based correlational analysis. This method evaluated the RSFC by quantifying the Pearson correlation coefficient of regionally averaged spontaneous BOLD signals between different brain regions. A linear mixed effect model was used to determine the overall effect on RSFC of each group of rats, while removing any random effect from different batches of animals.
